# A Reassessment of the Pseudoneglect Effect: Attention Allocation Systems Are Selectively Engaged by Semantic and Spatial Processing

**DOI:** 10.1037/xhp0000882

**Published:** 2020-12-03

**Authors:** Oliver J. Gray, Martyn McFarquhar, Daniela Montaldi

**Affiliations:** 1 Division of Neuroscience and Experimental Psychology, School of Biological Sciences, University of Manchester

**Keywords:** attention, semantic, lateralization, pseudoneglect

## Abstract

Healthy individuals display systematic inaccuracies when allocating attention to perceptual space. Under many conditions, optimized spatial attention processing of the right hemisphere’s frontoparietal attention network directs more attention to the left side of perceptual space than the right. This is the pseudoneglect effect. We present evidence reshaping our fundamental understanding of this neural mechanism. We describe a previously unrecognized, but reliable, attention bias to the right side of perceptual space that is associated with semantic object processing. Using an object bisection task, we revealed a significant rightward bias distinct from the leftward bias elicited by the traditional line bisection task. In Experiment 2, object-like shapes that were not easily recognizable exhibited an attention bias between that of horizontal lines and objects. Our results support our proposal that the rightward attention bias is a product of semantic processing and its lateralization in the left hemisphere. In Experiment 3, our novel object-based adaptation of the landmark task further supported this proposition and revealed temporal dynamics of the effect. This research provides novel and crucial insight into the systems supporting intricate and complex attention allocation and provides impetus for a shift toward studying attention in ways that increasingly reflect our complex environments.

## Public Significance Statement

This study describes a previously unrecognized but reliable spatial attention bias that is associated with the processing of the semantic meaning of objects. This counters the spatial attention bias well-known as the pseudoneglect effect. Our findings implicate a crucial role for the understudied left frontoparietal cortex in distributing attention, and open new, exciting areas for research. This work also reveals a mechanism that potentially enables our attention to be directed equally to different areas of space in daily life.

The frontoparietal attention (FPA) networks are specialized networks governing the distribution of attention to different areas of perceptual space (Mesulam, 1981). For many years, the right hemisphere FPA network has been considered dominant and optimized for this function (Heilman & Van Den Abell, 1980; Zuanazzi & Cattaneo, 2017). The *pseudoneglect effect* describes the tendency of healthy individuals to allocate more attention to the left side of perceptual space than the right (Bowers & Heilman, 1980). This behavioral effect has been putatively characterized as the behavioral manifestation of right FPA network optimization (Benwell, Harvey, et al., 2014; Jewell & McCourt, 2000; Zago et al., 2017).

**Figure 1 fig1:**
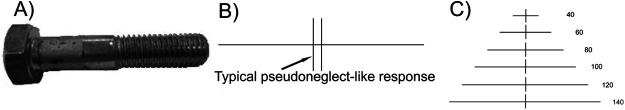
Example Objects and Lines Used in Experiment 1 *Note.* (A) An example object employed in the object bisection task. (B) A typical pseudoneglect-like response illustrated on a 160-pixel line. (C) The eight-line length types (20, 40, 60, 80, 100, 120, 140, and 160) assessed in the line bisection task with accurate central bisections.

The line bisection test (Reuter-Lorenz et al., 1990; Thiebaut de Schotten et al., 2005), the landmark and tachistoscopic forced-choice line bisection tasks (Benwell, Harvey, et al., 2014; Benwell, Thut, et al., 2014; McCourt & Jewell, 1999; McCourt & Olafson, 1997), and greyscale task (Nicholls & Roberts, 2002; Thomas et al., 2014), paradigms used to demonstrate the pseudoneglect effect, all require a participant to produce a stimulus centric comparison of the left and right sides of an image. For example, the line bisection task requires the participant to use a straight vertical line to perfectly bisect a straight horizontal line. Upon inspection of a centrally presented line, the horizontal extremities of the stimulus fall into the visual field that is processed by the contralateral hemisphere. Interestingly, healthy individuals show a tendency to allocate more attention toward the left side of visual space on these tasks (Benwell, Thut, et al., 2014; McCourt & Jewell, 1999; McCourt & Olafson, 1997; Nicholls & Roberts, 2002; Thiebaut de Schotten et al., 2005; Thomas et al., 2014). Most individuals will consistently place the vertical line to the left of the actual center of the horizontal line (see Figure 1). A traditional mechanistic account (Heilman & Van Den Abell, 1980) states that more efficient visuospatial processing of the left visual field by the right hemisphere’s FPA network increases the perceived size of the left side of the target. This size overestimation of the target of attention and/or a size underestimation of the nonattended section of the line results in a shift in the perceived center away from the midline to the left side (for a review of the pseudoneglect effect with the line bisection task, see Jewell & McCourt, 2000).[Fig-anchor fig1]

In revealing pseudoneglect, the line bisection, landmark, and greyscale tasks have helped to characterize the functional architecture of the FPA network in healthy individuals (Benwell, Harvey, et al., 2014; Learmonth et al., 2017; Szczepanski & Kastner, 2013; Thiebaut de Schotten et al., 2011) and establish the factors affecting allocation of attention (Jewell & McCourt, 2000; Learmonth et al., 2017). For example, variations in line length has been consistently shown to affect the extent of the bias on the line bisection task. The bisection of small lines is associated with generally more central/accurate judgments than longer lines, which most often elicit the typical leftward ‘pseudoneglect’ response (Benwell, Harvey, et al., 2014; Benwell, Thut, et al., 2013; McCourt & Jewell, 1999).

The line bisection and landmark tasks are used to provide an account of the processing of attention allocation that is unbiased by other cognitive processing (Jewell & McCourt, 2000). Allocating attention to judge the length of lines or the darkness of the shading in greyscale bars is very rarely required outside of a controlled scientific testing environment. Despite the ability to control confounding factors in lab-based experiments, substantial individual variability in lateralization bias is common (Learmonth et al., 2017; McCourt, 2001) and even our basic understanding of how pseudoneglect applies in complex and ecologically meaningful environments is limited (Darling et al., 2018; Hatin et al., 2012; Learmonth et al., 2018; Nicholls et al., 2007). For example, a recent study unexpectedly observed rightward biases in the preference of passengers choosing which side of an aircraft aisle to sit on (Darling et al., 2018). Similarly, others studying lateralization biases have observed that abstract, lab-based measures may be missing aspects of attention that are important in environmentally realistic scenarios (Learmonth et al., 2018). Understanding how attention allocation occurs in these environments and what neural processes make the most important contributions would enable better characterization of naturalistic attention allocation biases. Visual space in daily life almost always contains identifiable information and the identity of objects in visual space will, in many circumstances, automatically engage the processes dedicated to item identification or recognition. These processes and their interaction with attention allocation are investigated in this study.

Attention is guided by two distinct networks in each hemisphere. The dorsal attention network guides attention to highly specific areas of visual space (e.g., the intraparietal sulcus contains topographic maps of visual space, Swisher et al., 2007; Wang et al., 2015). In contrast, the ventral attention network is comprised of regions processing nonspatially specific functions (e.g., arousal and disengaging goal-directed attention). Although functional lateralizations have been identified in the dorsal attention network (Cai et al., 2013; Foxe et al., 2003), these lateralizations are thought to be highly dependent on an unequal drive of each dorsal network by each hemisphere’s ventral attention network (Chambers et al., 2004; Corbetta & Shulman, 2002, 2011; Thiebaut de Schotten et al., 2011). Lateralization of activity in the ventral system causes both the aberrant spatial attention associated with unilateral hemispatial neglect and the pseudoneglect bias in healthy individuals through its engagement of the spatially specific dorsal network via the superior longitudinal fasciculi (Corbetta & Shulman, 2002, 2011; Thiebaut de Schotten et al., 2011). This ventral drive of the dorsal system was effectively demonstrated by Thiebaut de Schotten et al., (2005) who showed that direct intraoperative electrical stimulation of the inferior parietal lobule and the superior longitudinal fasciculus caused lateral deviations on the line bisection task.

The inferior parietal lobule (IPL) is a key node of the ventral attention network. This is clear in cases of severe unilateral hemispatial neglect where patients with selective IPL damage cannot disengage from ipsilesional visual space (Hillis et al., 2005). Evidence from transcranial magnetic stimulation (Chambers et al., 2004, 2007; Petitet et al., 2015) has reinforced this prominent role of the IPL in driving the balance of dorsal attention network activity in each hemisphere and attention to visual space.

Processing in the left IPL has also been closely linked with semantic processing (Binder et al., 2005; Bonner et al., 2013; Seghier, 2013; Seghier et al., 2010; Vigneau et al., 2006). Indeed, a meta-analysis of neuroimaging contrasts, targeting different IPL functions reported strong evidence linking “automatic semantic processing” (e.g., reading words > nonwords, concrete > abstract words) with left, but not right, hemisphere angular gyrus activity (Humphreys & Lambon Ralph, 2015). In addition, the retrieval of semantic information from episodic memory has been closely linked to left, but not right, IPL activity (Gray et al., 2020) and the left angular gyrus has been identified as a semantic hub; reflecting its heteromodal response to semantic information (Bonner et al., 2013; Vigneau et al., 2006). The potential for interaction between semantic processing and systems that govern the allocation of attention has been suggested, but not effectively measured (Lee et al., 2004; Turriziani et al., 2009). Lee et al. (2004) observed that single, straight lines made up of unfamiliar letters (e.g., ㅂㄴㅂㄹㅂㅂㄴㅂㅂㄷㄷㅂㅁㅂㄱㅂㅋㅂㅋ) or multiple nonletter characters, (e.g., ▲♠★♦★♣▲♯♦▲★♯♦★♠♦▲♦), produced more central bisections than the traditional solid line bisection task. This suggests that the pseudoneglect effect may be disturbed or modulated by more complex information. Furthermore, it implies that the presence of semantically interesting features within objects may induce interaction between the language processing conducted by specialized regions in the left hemisphere and the left hemisphere FPA network. This potential has prompted us to hypothesize that object bisections will engage these same interactions and induce more rightward biased errors than line bisections.

Another investigation that utilized more complex and ecologically realistic stimuli focused on representational space and semantic concepts (Turriziani et al., 2009). Individuals were asked to make judgments based on the degree of semantic difference between a centrally presented image and images presented in the left and right hemifields. In this study, errors primarily originated from overestimation of semantic difference in the right hemifield (Turriziani et al., 2009). However, a within-subjects contrast with nonsemantic spatial judgments was not performed. This makes it difficult to assess the mechanistic distinction between these findings and previously observed pseudoneglect effects in attention. It is also unclear from this study whether semantics can affect perceptual attention allocation, or if the findings only reflect a bias in explicit conceptual judgments.

Development of a paradigm without an intrinsic directional arrangement (e.g., European languages read left to right), improved ecological validity, and comparability with traditional attention allocation tasks is critical to developing a better understanding of the intricacies of spatial attention allocation. To the authors’ knowledge, no study has assessed the effect of object processing on a task assessing lateralization bias in the allocation of attention until now. Object perception can induce semantic labeling, categorization, word association, semantic retrieval, and potentially cued recall of episodic memory. These functions have all been associated with IPL activity in the left hemisphere (Humphreys & Lambon Ralph, 2015; Vigneau et al., 2006). We utilized a variant of the traditional line bisection task in which participants moved a vertical line to centrally bisect an object (see Figure 1). The same individuals also performed a traditional line bisection task.

As well as potentially providing additional insight into the neural systems involved, we invoked several control measures and additional analyses to ensure that we could confidently assess the impact of semantic processing on attention allocation. Though the width of all objects was constant, the geometries varied across objects (e.g., taller, shorter, more pixels on the left/right side). The inclusion of analyses of these geometries also allowed for comparison with previous work highlighting the interaction between line geometry and attention allocation (McCourt & Garlinghouse, 2000). Participants bisected the horizontal mirror image of all objects to control for differences in geometry and object features between the left and right sides of the image. In the line bisection task, participants made bisections of lines of 8 different lengths to compare the well-established line length effect with object bisection data. We also collected data on the number of keystrokes made on each trial to provide a measure of motor engagement for each participant. Interaction between motor engagement and attention has been shown to affect bisection errors on the line bisection and landmark tasks (McCourt et al., 2001), however, whether this interaction is evident with more complex stimuli or is specific to simple abstract shapes has not been established.

To further assess the impact of semantic processing on attention allocation, Experiment 2 recruited a separate group of participants and adapted the computerized bisection task to assess the bisection errors associated with object-like abstract shapes. This approach removed the presence of features that automatically enable object recognition while matching the shape of the object’s outline in Experiment 1. We expected that these object-like shapes would specifically engage semantic processing more than line stimuli but less than objects.

Last, we used an object-based alternative to the traditional landmark task in Experiment 3. This assessed whether semantic processing would affect covert and overt attention allocation differently. In addition, this also provided a characterization of the temporal dynamics of the semantic and attention interaction.

## Experiments 1 and 2

### Method

#### Materials

The experimental session took place in a dedicated testing room at the University of Manchester. All visual tasks were completed on a desktop computer with a 17-in. monitor. Participants sat centrally in front of a desk. Participant’s eyes were approximately 72 cm from the screen, but head position was not fixed. A standard QWERTY keyboard was placed directly in front of the screen such that the number pad, used for all responses in Experiments 1 and 2, was in a comfortable position for our right-handed participants.

#### Procedure

Participants were instructed to move a peripherally presented vertical line (162 mm/∼12.84°) to bisect the target stimulus as close as possible to its center, defined as equidistance between the left and right edges of the stimulus. The experimenter indicated the vertical line and the edges of an example target stimulus in each block to ensure participant understanding. On the number pad of the keyboard, the numbers *4* and *6* moved the vertical line, left and right respectively, across the screen 4.5 mm (∼0.36°) per key press. Numbers *1* and *3* moved the vertical line, left and right, respectively, 0.45 mm (∼0.036°) at a time. To minimize motor demands, participants were encouraged to press and hold the keys to move the line smoothly. The side on which the vertical line was initially presented (left or right) was counterbalanced across trials. In addition, the original position on each side varied randomly within 20 mm (∼1.60°). All target stimuli (horizontal lines) were presented centrally onscreen. No time limit was set; instead, participants were instructed to be as accurate as possible and to move on to the next example as soon as they believed the bisection to be accurate. On average, participants required 5 s to 10 s to perform the bisection of each stimulus. To minimize task-switching effects, participants completed the line and object bisection tasks in separate blocks. Block order was counterbalanced across the participants in Experiment 1.

#### Data Analysis

All analyses were performed using linear mixed-effects models implemented in the lme4 package (Bates et al., 2015) in R (R Development Core Team, 2018). These models allowed us to include all data points from all trials in our analyses to substantially increase the power of our estimates (Brysbaert & Stevens, 2018). Our modeling approach was to specify a random slope and intercept structure for each model to allow for the most general marginal covariance structure possible, given the data. The most parsimonious structure was then discerned using likelihood ratio tests against more simple structures, as described in West et al., (2015). Omnibus tests of the model terms were conducted using asymptotic Wald’s chi-square tests, as implemented in the Anova function from the car package (Fox & Weisberg, 2011). Explorations of significant omnibus effects were conducted using the phia package (De Rosario-Martinez, 2013) with Holm’s method used to correct for multiple comparisons (Holm, 1979). Extraction and plotting of the various model effects was conducted using the effects package (Foxe et al., 2003) and ggplot2 (Wickham, 2016). We provide descriptive statistics detailing mean-differences and regression slope coefficients to aid the interpretation of our results.

## Experiment 1: Object and Line Bisection Tasks

### Participants

A total of 26 (one male; age range = 18–35 years, *M* = 19.73) participants with normal or corrected-to-normal vision, and no history of neurological disorder completed the object and line bisection variants of the task. Estimations of power in linear mixed effects models are both complex and debatable (Brysbaert & Stevens, 2018; Judd et al., 2017) and the absence of established techniques prompted us to base our sample size on that of similar investigations (Benwell, Harvey, et al., 2014; Benwell, Thut, et al., 2013) that observed significant differences in bisection biases between different stimulus types using similar methods of analysis. All participants read the participant information sheet and provided written informed consent at the start of the experimental session. All procedures were approved by the University of Manchester Research Ethics Committee.

### Images

A total of 144 images were presented in the present study. Straight black horizontal lines (72) measuring a width of 9 mm (approximately 0.72° visual angle), 18 mm (∼1.43°), 27 mm (∼2.15°), 36 mm (∼2.86°), 45 mm (∼3.58°), 54 mm (∼4.30°), 63 mm (∼5.01°), and 72 mm (∼5.73°) were presented in the line bisection task.^1^ Individual greyscale photograph images of everyday, unrelated objects (72 images) developed by Frank et al., (2019) and Migo et al. (2013) were presented in this experiment. All object images measured 76 mm (∼6.042°) from left to right. Vertical image size varied between 9 mm (∼0.72°) and 122 mm (∼10°). Participants also bisected the mirror image of all objects to control for the inherent horizontal asymmetry of both the shape and position of distinctive features of many objects. Stimuli were presented and responses recorded through E-Prime 2.0 (Schneider et al., 2012).

## Experiment 2: Object-Like Abstract Shape Bisection

### Participants

A further 33 (5 males, 18–35, *M* = 21.03) participants with normal or corrected-to-normal vision and no history of neurological disorder completed the bisections of object-like abstract shapes. All participants read the participant information sheet and provided written, informed consent at the start of the experimental session. All procedures were approved by the University of Manchester Research Ethics Committee.

### Images

Object-like abstract shapes (72) were developed by using the object images developed by Migo et al., (2013) as a template and removing all internal image features by completely filling the shape with gray color (RGB = 102). Edges that clearly revealed the original identity of the shape were then removed by the addition of a small filled gray rectangle. This procedure resulted in a set of stimuli that were not easily recognizable but retained most of the spatial characteristics of the original image (see Figure 2). The ease/difficulty with which one could interpret the object identity of a shape was assessed in a separate group of participants. Participants in this shape interpretation rating experiment were shown all shapes and asked to provide a rating of how easy/difficult it was to interpret the object identity of the image. We found in this experiment that, generally, participants ratedthe shapes *difficult* or *very difficult* to interpret as objects. The procedure and distribution of results is presented in Supplementary Materials 1. As in Experiment 1, the mirror image of all the shapes was also presented in Experiment 2 to control for the inherent horizontally asymmetrical features of the shape.[Fig-anchor fig2]

## Results

### Experiment 1: Object and Line Bisection

#### Line Bisection

**Figure 2 fig2:**
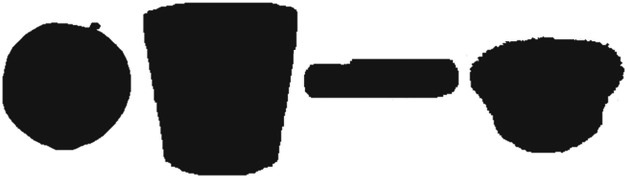
Four Examples of Shapes Employed in the Object-Like Abstract Shape Bisection Task

**Figure 3 fig3:**
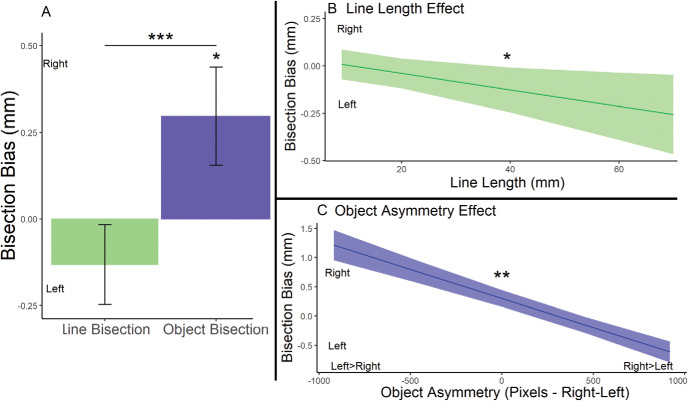
A Summary of Data From the Object and Line Bisection Tasks in Experiment 1 *Note.* (A) An illustration of the mean bisection errors of the line and object bisection tasks. We observed a significant difference between the direction of the mean bisection errors associated with lines and objects (all line lengths***, shortest lines*). In addition, object bisection errors were bisected significantly to the right of the objects’ center*. (B) An illustration of the significant association between greater line lengths and increasingly leftward bisection errors*. (C) An illustration of the significant** relationship between the object asymmetry and bisection errors. Objects with right > left displayed more rightward bisection errors. Conversely, objects with left > right displayed more leftward bisection errors. Negative bisection errors depict leftward bisection errors, and positive bisection errors depict rightward bisection errors. * *p* < .05. ** *p* < .01. *** *p* < .001.

We began by performing a comparison of line bisection errors without distinguishing between the different line lengths (*M* = leftward by 0.131 mm^2^, *SE* = 0.12 mm^2^) against zero (100% accuracy/unbiased responding). This comparison revealed no significant difference from zero, χ^2^(1) = 1.30, *p* = .25 (see Figure 2). We subsequently assessed the effect of line length (defined as a continuous variable: 9 mm, 18 mm, 27 mm, 36 mm, 45 mm, 54 mm, 63 mm, 72 mm) and number of keystrokes on line bisection errors. As predicted, we observed a significant main effect of line length, χ^2^(1) = 4.14, *p* = .04. This effect described the association between longer lines and more leftward bisection errors, commonly known as the line length effect (see Figure 3, slope estimate = more leftward by 0.8 mm for a 1 cm increase in line length, *SE* = 0.4 mm).[Fig-anchor fig3]

We also observed a trend suggesting an interaction between line length and keystrokes, χ^2^(1) = 3.55, *p* = .06. However, due to the difficult nature of investigating an interaction between two continuous variables, and the nonsignificant nature of the interaction in question, we did not perform further formal analysis of this effect. We did not observe a significant main effect of keystrokes on bisection errors, slope estimate = 0.03 mm rightward/1 cm, *SE* = 1.29 mm, χ^2^(1) = 0.001, *p* = .97.

#### Object Bisection and Differences From Line Bisection

To investigate the bisection errors associated with object images, we compared these errors with zero (100% accuracy/unbiased responding). Interestingly, we observed errors in the bisection of objects (*M* = rightward by 0.30 mm, *SE* = 0.14 mm) that were significantly rightward of center, χ^2^(1) = 4.40, *p* = .04 (see Figure 3). We then performed a within-subjects assessment of whether object bisection errors were significantly different to line bisection errors (collapsed across all line lengths). We found that object bisection errors were significantly more rightward than line bisection errors, χ^2^(1) = 9.81, *p* = .002. As mentioned, we observed the most central/rightward bisections with lines of a shorter length. Small lines have also previously been shown to produce the most rightward bisection errors (Benwell, Harvey, et al., 2014; McCourt & Jewell, 1999). Critically, we observed significantly, χ^2^(1) = 6.91, *p* = .01, more rightward bisections of objects than the smallest lines (9 mm—very similar to the line length used by Benwell et al., 2014; *M* = leftward by 0.15 mm).

Our final within-subjects analysis assessed the impact of horizontal asymmetry on object bisection errors (see McCourt & Garlinghouse, 2000 for an assessment of horizontal asymmetry with line stimuli). We established the size difference between the left and right sides of each object image (number of pixels). We then assessed whether this difference (*SD* = 462.7 pixels, range = −1026–1026) and/or the number of keystrokes were associated with changes in bisection errors. We did not observe a significant effect of number of keystrokes (*M* keystrokes used = 7.5, *SD* = 4.8) on bisection errors, slope estimate *=* leftward by 0.05 mm/25 keystrokes, *SE* = 0.07 mm, χ^2^(1) = 0.47, *p* = .47. The degree to which an image was nonsymmetrical was associated with significant changes in bisection errors, slope estimate = leftward by 0.1 mm/100-pixel difference between left and right, *SE* = 0.015 mm, χ^2^(1) = 27.97, *p* < .001. This illustrated that objects with more pixels on the right than the left side displayed more leftward biases. Conversely, more rightward bisection errors were associated with objects with more pixels on the left side of space (see Figure 3). We did not observe any interaction between motor engagement and the degree to which an object was nonsymmetrical, χ^2^(1) = 0.002, *p* = .97. These findings are consistent with those observed with horizontally asymmetrical line stimuli in McCourt and Garlinghouse (2000).

### Experiment 2: Object-Like Abstract Shape Bisection

#### Bisection Errors in Shape Bisection

**Figure 4 fig4:**
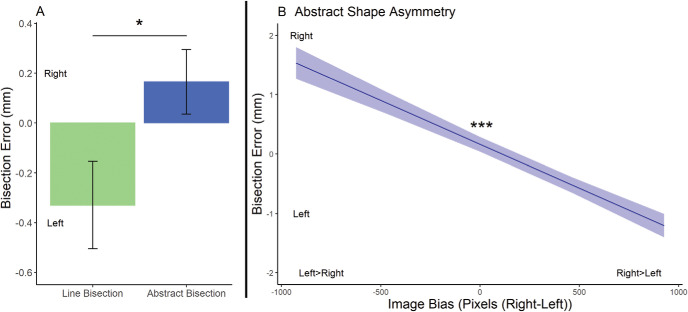
A Summary of Data From the Object-Like Abstract Shape Bisection Task in Experiment 2 *Note.* (A) We observed shape bisection errors that were not significantly different from center or all line lengths but were significantly rightward of lines of a similar length to the shapes*. (B) An illustration of the significant*** relationship between object asymmetry and bisection errors. Shapes with right > left displayed more rightward bisection errors. Conversely, shapes with left > right displayed more leftward bisection errors. Negative bisection errors depict leftward bisection errors, and positive bisection errors depict rightward bisection errors. * *p* < .05. *** *p* < .001.

As in Experiment 1, we first assessed the difference between the participant bisection errors in the object-like abstract shapes bisection task (*M* = 0.17 mm, *SE* = 0.12 mm) and zero/central bisection. Errors in the bisection of shapes were not significantly, χ^2^(1) = 1.90, *p* = .17, different from center (though note the rightward direction of the mean bisection errors in our sample; see Figure 4).[Fig-anchor fig4]

Like Experiment 1, we then assessed whether horizontal asymmetry of the image and motor engagement levels (measured by number of keystrokes; *M* keystrokes used = 9.4, *SD* = 5.3) affected the direction of bisection errors. This revealed a significant effect of horizontal asymmetry, slope estimate = 0.15 mm/100-pixel difference between left and right, *SE* = 0.02 mm, χ^2^(1) = 47.09, *p* < .001, and a significant effect of keystrokes on bisection errors, slope estimate = rightward by 0.24 mm/25 keystrokes, *SE* = 0.1 mm, χ^2^(1) = 5.64, *p* = .02. As in Experiment 1, the horizontal asymmetry effect describes the linear association between greater leftward bisection errors and the increasingly rightward bias in pixel ratio between the right and left side of space (see Figure 4). More keystrokes were associated with less leftward/increasingly rightward bisection errors. We observed no significant interaction between horizontal asymmetry and motor engagement, χ^2^(1) = 0.10, *p* = .75.

#### Differences Between the Bisection of Object-Like Abstract Shapes and Lines

We also assessed whether shape bisection errors were different to the line bisection errors measured in Experiment 1. We did not observe a significant difference between shape and line bisection errors when we collapsed across all line lengths, χ^2^(1) = 12.36, *p* = .14. Though not significant, our sample mean for bisection errors was to the right of center and therefore the use of smaller lines for the line bisection task may have impacted this comparison. The significant effect of line length in Experiment 1 (longer lines were associated with more leftward bisection errors) prompted us to compare lines and object-like abstract shapes with a comparable length (width = 63 mm and 72 mm). Here, we observed significantly more rightward bisection errors associated with shapes than with lines, χ^2^(1) = 5.17, *p* = .02.

## Interim Discussion—Experiments 1 and 2

### The Attention Allocation Systems Are Selectively Specialized for Either Spatial or Semantic Processing

As it is well established that the bisection of simple lines readily generates leftward bisection errors (Jewell & McCourt, 2000), this experiment aimed to assess the effect of increased target complexity on bisection errors in healthy individuals and improve our understanding of how the effect could generalize across more realistic everyday stimuli. The processing of objects is extremely common in daily life. Our interactions with them are both more frequent and varied than those we have with more abstract shapes or lines. Like words, the semantic processing of objects (e.g., labeling them, categorizing them, retrieving related information from memory) has often been associated with IPL activity lateralised to the left hemisphere (Devereux et al., 2013; Gray et al., 2020; Vigneau et al., 2006). Experiments 1 and 2 explore how spatial attention interacts with the semantic processing of objects.

In Experiment 1, the bisection of objects was associated with errors that were significantly rightward of both central, completely accurate responding, and the errors that were associated with bisection of line stimuli. Line bisection errors were not significantly leftward of center when collapsed across line lengths, however, our results replicated the well-established line length effect, whereby increases in line length are accompanied by greater leftward bisection errors (see Figure 3; Benwell, Harvey, et al., 2013, 2014; McCourt & Jewell, 1999). Critically, we observed errors associated with bisection of objects that were significantly rightward of even very small lines (i.e., the most likely to produce central/rightward bisection errors; Benwell, Harvey, et al., 2014; McCourt & Jewell, 1999). Our results show that the rightward bias in the bisection of objects is distinct from a bias one could expect from the smallest lines. Instead, this difference suggests that the attention bias is modulated by a different factor. These results fit with the idea that semantic processing in the left FPA network could cause a rightward bias in attention allocation.

Experiment 2 further assessed the contribution of the semantic aspect of object processing to the right visual field bias observed in Experiment 1. In the current study, objects were not mentioned by the experimenter prior to or during the shape bisection session. Despite this, when debriefed, all participants reported interpreting many of the shapes as objects. Although the identity of each item being task-irrelevant, the identification of one shape as an object may have encouraged participants to attempt (intentionally or unintentionally) to interpret the subsequent object-like abstract shapes as objects. Interestingly, we observed evidence that our object-like abstract shapes displayed a bisection bias between those associated with lines and objects. Namely, bisections of object-like abstract shapes were significantly rightward of line bisection errors of an equivalent width. However, this was not evident when shorter lines (associated with more central bisection errors—Experiment 1) were included in the analysis. This is unlike objects, which displayed a more rightward bias than even the shortest lines.

Our findings using object-like abstract shapes are in stark contrast to the findings of Pia et al. (2010) and previous work (Aniulis et al., 2016; Churches et al., 2017) using semantically unengaging geometric shapes (circle, rectangle, triangle, Rey-Osterrieth complex figures).These studies revealed consistent, and frequently significant, evidence of a leftward bisection bias. Pia et al. (2010) asked participants to bisect a line drawing image of a basset-hound stretched so it resembled two parallel line stimuli with meaningful information at either end. Participants bisected only this stimulus 120 times presented in different orientations throughout the experiment, demonstrating a leftward visual attention bias. Although the stimulus may have been initially semantically engaging, this information was not relevant to the bisection task and thus, participants are likely to have habituated to, or even ignored, the information after a few trials. As a result, these findings provide no specific insight into the interaction between semantic processing and spatial attention allocation. Next, where one specific type of semantically minimalist shape has not displayed a leftward bisection bias (e.g., a square), the sample mean has not before been measured to the right of center, as is observed here. We propose that our finding of a complete lack of evidence of a leftward bias with object-like shapes and their significant difference from line stimuli of the same width provides additional evidence for the role for semantic processing in driving a rightward bias in attention. This is evident in the progressively more rightward bisection errors that are associated with the increase in semantic processing from lines to object-like abstract shapes to objects.

To summarize, we have observed rightward bisection errors that are specific to objects and object-like stimuli. As a large and consistent rightward bisection bias is indicative of greater activation of the left hemisphere FPA network (Kinsbourne, 1970; Loftus & Nicholls, 2012), these findings provide strong, novel evidence that the semantic processing associated with object recognition can induce a rightward bias in attention allocation. Furthermore, it suggests a balance of processing that is more complex than the traditional account that the right hemisphere’s FPA network is uniquely specialized for attention allocation. Although fully comprehending this processing will require studies utilizing neuroimaging and/or neurostimulation, it is likely that the interaction between semantic processing and attention allocation occurs within the left hemisphere’s FPA network. The IPL represents one brain region that may produce this interaction. In the right hemisphere, the IPL has been causally linked with highly perceptual and spatial processing and is one driver of the pseudoneglect effect (Chambers et al., 2004, 2007; Petitet et al., 2015). In contrast, the IPL in the left hemisphere has been closely linked with semantic judgments (Davey et al., 2015; Humphreys & Lambon Ralph, 2015; Seghier et al., 2010; Vigneau et al., 2006). Indeed, left IPL activation is observed in fMRI contrasts between concrete and abstract words (Binder et al., 2005), and real words and nonwords (Bonner et al., 2013). It is tempting to draw comparisons between these results and the bisection errors associated with object images, object-like abstract shapes, and lines in the current study.

### Insights From Patterns of Motor Engagement

While making bisections of all stimulus types, participants made multiple keystrokes to move the vertical, laterally presented bisecting line to the center of the target. Evidence suggests that the motor system may interact with the FPA networks during attention allocation (Jewell & McCourt, 2000). First, the lateralization bias of attention allocation has been found to differ between left and right-handed individuals (Luh, 1995). Second, the use of the left or right hand to perform line bisection has been shown to modulate the attention bias (Brodie & Pettigrew, 1996; McCourt et al., 2001). This evidence prompted us to assess the impact of motor engagement across our analyses of line, object, and object-like abstract shape bisection errors to ensure that motor effects could not explain differences between lines, objects, and shapes. We also sought to gain better insight into the influence of motor engagement on the allocation of attention.

In the line bisection task, we observed a trend suggesting an interaction between the bisection errors associated with different line lengths and the keystrokes used for bisection. Though we did not conduct formal analysis of this interaction trend (partly because of the difficulty of assessing interactions between two continuous variables), visual inspection did suggest that less leftward/more rightward bisection errors were associated with the use of more keystrokes in the longer line lengths. We suspect that the assessment of shorter, as well as longer lines reduced our ability to detect motor effects in this task more clearly.

In contrast with the line bisection task, the object bisection task showed no evidence of changes in bisection errors with differing levels of motor engagement. This is despite the objects being of a similar width to the longest lines in the line bisection task, which themselves were potentially the most sensitive to motor engagement. We propose that the left hemisphere’s FPA network is engaged during the object and object-like abstract shape tasks but not the line bisection task. We suggest that right-handed motor engagement can increase the activity of the LPA network in a lower activity state (the line bisection task). However, a ceiling effect may prevent the same effect of motor engagement on the left FPA network in a state of higher activity. This may prevent additional rightward deflections on bisection errors associated with higher motor engagement in the object bisection task.

We suggested in the preceding text that our object-like abstract shapes recruit more semantic processing than lines, but not as much as objects. Consequently, they were associated with bisection errors that were significantly different from width-matched lines, but not significantly different from center. Interestingly, we observed significantly less leftward/more rightward bisection errors accompanying the use of more keystrokes for bisection. This is in-line with our assessment of the nature of the motor engagement effect characterizing the interaction trend in the line bisection task, and our supposition that object-like abstract shapes induce a hemispheric balance of attention between those elicited by lines and those elicited by objects.

### Object and Shape Asymmetry

Our analyses of the impact of object and shape asymmetry revealed significant effects of horizontal image asymmetry on bisection errors. This observation mirrors that of McCourt and Garlinghouse (2000) who demonstrated that an image with a strong horizontal bias elicited bisection errors that were biased away from the largest side of the image. In contrast to Experiments 1 and 2, this study presented only triangular wedges (▶ or ◀) and observed a pseudoneglect effect. Despite the clear difference in laterality of the overall attention bias, the current study has demonstrated that the effect of stimulus geometry is pertinent in a large sample of images of everyday objects and shapes that have not been stretched or modified. Moreover, we have shown that the extent of image asymmetry influences the lateralization of bisection errors. In other words, bisection errors are influenced by image asymmetry and not simply in a binary fashion reflecting left > right or right > left.

### Covert Attention Allocation and Semantic Processing

Pseudoneglect has also been observed in studies utilizing versions of the landmark task that assess covert attention allocation (Benwell, Harvey, et al., 2014; Learmonth et al., 2017; Learmonth, Gallagher, et al., 2015; McCourt & Olafson, 1997). The traditional line, object, and shape bisection tasks, and the previous investigations that hinted at a semantic-attention interaction (Cristescu et al., 2006; Turriziani et al., 2009) all allow time for overt shifts in attention and deliberate comparisons across the two hemifields. In contrast, the allocation of covert attention occurs before/without the movement of the eyes. Observation of pseudoneglect with covert attention shifts using tachistoscopic stimulus presentations demonstrated that the effect was not caused by systematic or biased scanning of stimuli by the eyes (McCourt & Olafson, 1997). In this case, the assessment of covert attention enabled a better assessment of which functions and neural systems produced the pseudoneglect effect. We adopted the same approach in Experiment 3 to gain better insight into the findings presented thus far.

With semantically unengaging stimuli, participants only need to direct their attention to make the bisection judgment. In contrast, object bisections will be associated with both semantic processing and the direction of attention. The behavioral manifestations of semantic processing may occur more slowly than the direction of attention. This semantic delay may reduce or prevent overlap of these manifestations with the fast processing of covert attention allocation. More simply, an individual may covertly attend before additional semantic processing can exert a measurable influence. This case would impact predictions of which brain regions supported the effect and the future questions to be investigated.

Experiments 1 and 2 have revealed evidence of an interaction between the systems governing the allocation of attention and the semantic processing accompanying object recognition. This striking interaction illustrates the narrow focus of the traditional line bisection task on visuospatial processing. Given the discovery of the impact of semantics on attention allocation, the ecological applicability of the pseudoneglect effect should be considered carefully. The way each FPA network drives the allocation of attention to encounters that are more complex than observing straight lines or greyscales, requires substantial further investigation. In Experiment 3, we attempted to replicate our demonstration of the semantic-attention interaction that was observed in Experiments 1 and 2, and further investigate the effect using an adapted landmark task that taps both overt and covert attention allocation.

## Experiment 3: Comparing Semantic Processing in Overt and Covert Attention Allocation

We developed an object-based alternative to the traditional landmark task to investigate whether the attention-semantic interaction identified in Experiments 1 and 2 is also observable in covert attention allocation. The traditional landmark task involves making left, center, or right decisions regarding the position of a vertical transecting line, relative to the center of a horizontal line. As with the line bisection task, healthy individuals tend to estimate the left side of a horizontal line as longer than the right. This results in these participants reporting that the transecting line is further right than its objective position (i.e., bisections that are objectively positioned to the left of center are reported as central or rightward of center; Reuter-Lorenz et al., 1990). This has been interpreted as reflecting the optimization of the right FPA network for the covert allocation of attention to visual information.

We hypothesized that semantic processing systems would be recruited upon presentation of an object-vertical line display and that semantic processing, specialized in the left hemisphere, would induce activity in the left hemisphere’s FPA network. This activity would produce faster detection of subsequently presented targets in the right visual field. Critically, we also hypothesized that this semantic processing, and induction of left hemisphere FPA network activity would take longer than its purely visuospatial counterpart. As a result, with our novel object-landmark task, we expected to observe a strong reversed pseudoneglect effect in a free viewing (overt) condition, and the same effect to a lesser extent or no effect in a covert condition. This task required only one keystroke per trial (to indicate their decision choice) and thus minimized the effect of motor engagement observed in Experiment 1 (7.5 keystrokes/trial). As well as assessing accuracy of participant response, we included analysis of RTs to better assess the temporally dependent processes described in this section.

### Method

#### Participants

A separate group of 29 participants (15 male; age range = 18–35 years, *M* = 22.86) with normal or corrected-to-normal vision and no history of neurological disorder completed Experiment 3. All participants read the participant information sheet and provided written, informed consent at the start of the experimental session. All procedures were approved by the University of Manchester Research Ethics Committee.

#### Materials and Stimuli

**Figure 5 fig5:**
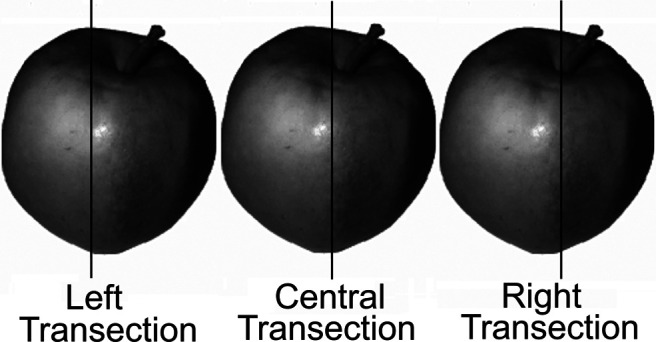
Examples of Stimuli That Were Presented in the Object–Landmark Task *Note.* Participants were asked to indicate whether the vertical line was in the center of the object or positioned to the left or right of center.

The equipment used in Experiment 1 and 2 was also used in Experiment 3. We used a black fixation cross measuring 21 mm × 21 mm with 4-mm thick lines to centrally fixate participants. The same objects from Experiment 1 were presented in Experiment 3. All objects were pretransected by a vertical line of the same dimensions as those used in Experiment 1. The transecting line was either exactly in the center of the object (28 trials) or to the left or right of center (84 trials each). Figure 5 provides examples of transected objects used in Experiment 3. Of those objects transected to the left or right of center, transections diverged by −7, −5, −3, 3, 5, and 7 pixels from center (28 trials each). All object images were presented with the transecting line in the center of the screen.[Fig-anchor fig5]

#### Procedure

Participants were instructed to fixate before each trial on a centrally presented cross and to use their peripheral vision to observe the images presented to them. An object was then presented to the participant for either 150 ms (fast enough to prevent observation through reactive eye movements; McCourt & Jewell, 1999; Szczepanski & Kastner, 2013) or 2,000 ms. Participants were instructed to indicate whether the transecting line was in the horizontal center of the object or whether it was to the left or right of center. Participants were required to respond within 2,000 ms of stimulus presentation irrespective of the image’s presentation time. Responses were made using the *1* (left of center), *2* (central), and *3* (right of center) keys on the keyboard number pad.

#### Data Analysis

The data analysis for Experiment 3 followed the same general approach used in Experiments 1 and 2. Again, all analyses were performed using linear mixed-effects models and the most parsimonious random-effects structure was discerned using likelihood ratio tests. The main difference from Experiments 1 and 2 was the use of a generalized linear mixed-effects model to assess the probability of correct identification of the object transection lateralization. This was achieved using the glmer function from the lme4 package with binomial errors and the logit link function (Bates et al., 2015).

### Results

#### Accuracy and the Side of Object Transection

**Figure 6 fig6:**
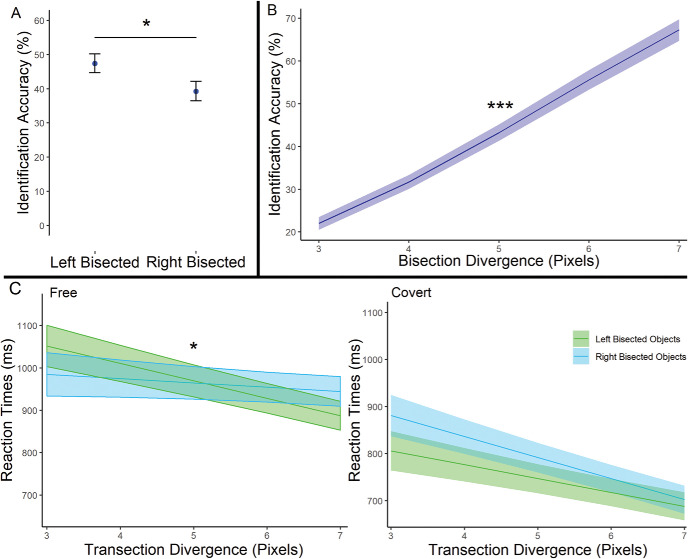
A Summary of Data From the Object-Based Adaptation of the Landmark Task in Experiment 3 *Note.* (A) We observed significantly* greater accuracy for identification of left transected than right transected objects. (B) We observed significant increases in accuracy accompanying greater transection divergences from center***. (C) An illustration of the significant* three-way interaction characterizing RTs. Note the significant* difference between the transection divergence slopes associated with left and right transected objects in the free viewing condition. This difference was not evident in the covert condition. The transection divergence slopes of right transected objects across the free and covert conditions were also significantly* different. This difference was not observed for left transected objects. Error bars represent one standard error. * *p* < .05. *** *p* < .001.

We first assessed whether the probability of correct identification of the object transection lateralization varied according to (1) the side of object transection (left/right), (2) the degree of transection divergence (3, 5, or 7 pixels), and/or (3) viewing condition (whether the stimulus was freely viewed or judged covertly). In accordance with our prediction, we observed a significant effect of the side of object transection, χ^2^(1) = 4.09, *p* = .04, such that accuracy was better for left transected objects (*M* = 47.43%) than right transected objects (*M* = 39.28%). We also observed a significant increase in accuracy accompanying increases in transection divergence from center, slope estimate = 9% increase/pixel increase in divergence, *SE* = 5%, χ^2^(1) = 91.77, *p* < .001. These results are displayed in Figure 6. We did not observe a significant effect of type of viewing condition, χ^2^(1) = 0.31, *p* = .58, or any interactions among the conditions: Transection Divergence × Side, χ^2^(1) = 2.62, *p* = .11, Transection Divergence × Viewing Condition, χ^2^(1) = 0.58, *p* = .45, Side × Viewing Condition, χ^2^(1) = 0.37, *p* = .54, Transection Divergence × Side × Viewing Condition, χ^2^(1) = 1.21, *p* = .27.[Fig-anchor fig6]

#### Reaction Times and the Side of Object Transection

We subsequently investigated the differences in reaction times (RTs) of correct responses using the same predictor variables as the analysis of accuracy. This analysis revealed a significant three-way interaction between the side of transection (left/right), degree of transection divergence, and viewing condition (free/covert), χ^2^(1) = 6.54, *p* = .01. The results of our subsequent analyses of the above three-way interaction are illustrated in Figure 6.

Our subsequent analyses of the three-way interaction explored whether the RT associated with increasing transection divergences, was different across the left and right transected images and the viewing conditions (covert/free). We observed that (1) the change in RT with increasing transection divergence of the right transected images (slope estimate = 10 ms faster/one-pixel increase in transection divergence, *SE* = 41 ms) was significantly different to the transection divergence slope for left transected images (slope estimate = 41 ms faster/one-pixel increase in transection divergence, *SE* = 40 ms) in the free viewing condition, χ^2^(1) = 6.22, *p* = .03. This suggested that participants did not gain the same RT advantage from the increase in transection divergence for right transected images as they did for left transected images. (2) This difference was not seen, χ^2^(1) = 1.39, *p* = .24, in the covert viewing condition (right transected images - slope estimate = 45ms faster/1-pixel increase in transection divergence, *SE* = 34ms; left transected images = 29ms faster/1-pixel increase in transection divergence, *SE* = 33ms). (3) We also revealed a significant difference between the RT slope of the right transected images across the free (10 ms/pixel) and covert (44 ms/pixel) viewing conditions, χ^2^(1) = 6.89, *p* = .02. (4) This significant difference was not present in the comparison of the RT slopes of left transected images across the free (as indicated earlier, 41 ms/pixel) and covert (as indicated earlier, 29ms/pixel) conditions, χ^2^(1) = 0.94, *p* = .38. Points 3 and 4 illustrate the specific lack of advantage gained from increases in transection divergence by right transected images in the free viewing condition.

### Interim Discussion—Experiment 3

The object-based adaptation of the landmark paradigm requires the participant to allocate attention to each side of an object and identify the lateralization of the line that is transecting it. The landmark task provides a sensitive measure of which side of visual space receives the preferential allocation of attention while mitigating any potential impact of motor engagement. Experiment 1 and 2 led us to expect that the interaction between semantic processing of objects and the left hemisphere’s FPA network would drive attention allocation preferentially to the right side of visual space. This would result in more instances of right transected images being identified as centrally or left transected images. In contrast, left transected images would be identified with greater accuracy and confidence. We have observed these predicted results throughout our analyses of the object-based landmark task to successfully replicate our demonstration of a semantic-attention interaction.

To summarize, we consistently observed evidence of accuracy advantages for objects with left sided transections. In contrast, right sided transections showed lower accuracy or weaker benefits associated with other factors. For example, we observed significantly greater left transection than right transection accuracy in the transection divergence analysis. In addition, we observed that increasing the divergence of transections from center was beneficial to RTs for left but not right transected images (see Figure 6). These effects highlight that attention allocation was consistently and preferentially allocated to the right side of visual space in the object-landmark task.

These results are in stark contrast with previous investigations using the traditional landmark task. The line-based landmark task is typically associated with the pseudoneglect effect, namely the preferential attention allocation to the left visual field. This results in greater lateralization identification accuracy and speed for right transected lines (Benwell et al., 2015; Benwell, Thut, et al., 2013; McCourt & Olafson, 1997). However, we show here that engaging semantic processing with objects produces greater identification accuracy and faster RTs to left transected images. These findings represent a complete reversal of the pseudoneglect effect. As with manual line bisection tasks, left visual field processing advantages in the landmark task has been linked closely with greater engagement of the right hemisphere’s FPA network (Benwell, Harvey, et al., 2014; Kinsbourne, 1970; Learmonth et al., 2017; Learmonth, Thut, et al., 2015; Reuter-Lorenz et al., 1990). It is therefore likely that the right visual field advantage observed here in the object-based adaptation of the landmark task reflects greater engagement of the left hemisphere’s FPA network.

In this experiment, we were also interested in the differences characterizing attention allocation under free viewing conditions compared with covert viewing conditions. We hypothesized that covert attention allocation may occur before additional, slower semantic processing can impact the balance of the FPA networks across the hemispheres. This would limit the degree to which the rightward attention bias can be elicited in a covert viewing condition. In Experiment 3, we observed a rightward attention bias in the free viewing condition in all relevant contrasts. In contrast, this rightward bias was observed only in the accuracy contrasts for the covert viewing condition. In summary of the RT results, we observed a difference in the RT advantage gained from bigger transection divergence for left and right transected object images in the free viewing condition. In contrast, we did not observe a significant difference between the transection sides in the covert condition (see Figure 6). A participant’s response could change after the presentation of a stimulus in the covert condition (presentation = 150 ms, *M* response RT = 780 ms) potentially allowing for semantic processing occurring after 150 ms to still affect the response and accuracy of the participant’s judgment. In contrast to the accuracy contrasts, the assessments of RTs are sensitive to the slower responses induced by this additional processing. As a result, the difference between the viewing conditions in reactions times suggests that the rightward bias was more easily observed in the free, than the covert, viewing condition. These results are consistent with our hypothesis about delayed semantic processing and limited rightward attention biases in the covert condition.

## General Discussion and Concluding Remarks

In this series of experiments, we have demonstrated for the first time that the hemispheric asymmetries of our attention allocation systems are specialized for both semantic and spatial processing. Our novel variant of the manual line bisection task has revealed a previously unrecognized but reliable perceptual effect, whereby the left hemisphere, specialized for semantic processing, has an attention allocation system that is optimized for, and selectively recruited by, semantically engaging stimuli. This recruitment results in a perceptual bias that is characterized by a greater engagement of attention with the right side of visual space. In addition, our object-based alternative to the landmark task has revealed differences in free viewing and covert viewing conditions, that provide insight into the temporal dynamics of the neural mechanisms supporting the interaction between semantic processing and attention allocation.

We have demonstrated here that semantic processing accompanying object bisection judgments can modulate the allocation of attention and produce a bias to the right visual field. This lateralization bias is distinct from attention allocation during a visuospatial challenge in the absence of semantic processing (as described by the traditional pseudoneglect phenomenon). As mentioned in the Introduction, the lateralization bias associated with the line bisection and landmark tasks has been linked closely with the activity of each hemispheres FPA network. In the absence of neuroimaging data, but in line with previous work and interpretations (Benwell, Harvey, et al., 2014; Foxe et al., 2003; Kinsbourne, 1970; Petitet et al., 2015; Reuter-Lorenz et al., 1990; Zago et al., 2017), our results suggest that using semantically engaging stimuli with these tasks engages the left FPA more than the right FPA.

The processing of object semantics involves their labeling and categorization, and the associative retrieval of semantically related information from memory. Our current data does not allow us to discriminate between the effect of these component processes, however, our findings that object-like abstract shapes drive the bias to the right side of visual space demonstrates that the success of object naming and classification, at least, are not critical to drive the effect. Instead, any attempt to engage these object-based semantic processes (independent of their success) may well generate a semantic cognitive state that is sufficient to trigger a shift in hemispheric balance and produce the right visual field attention bias. Future work should investigate this shift in balance with network-based neuroimaging assessments linked to specific behavioral measurements of the components of semantic processing.

We speculate that the IPL is a very promising candidate to drive the reversed pseudoneglect effect observed here. As described, the IPL upregulates activity in the spatially specific dorsal attention network (Corbetta & Shulman, 2011; Hillis et al., 2005; Thiebaut de Schotten et al., 2011). As a result, our findings could reflect the tight link between semantic processing and the left IPL (Gray et al., 2020; Humphreys & Lambon Ralph, 2015; Seghier, 2013; Seghier et al., 2010; Vigneau et al., 2006) that disproportionately increases left FPA network activity and biases the allocation of attention to the right side of visual space. This mechanism should be further explored using neuroimaging and neurostimulatory investigations.

The work presented here provides the first evidence of a semantic attention allocation system prioritizing information in the right side of space. New research that investigates the specific characteristics of the left hemisphere’s FPA network is now required to provide insight into the semantic attention allocation system, its role and dynamics, and its interaction with other factors, like item geometry and motor engagement, that modulate attention allocation in the absence of meaningful semantic information. Further work should seek to develop adaptable paradigms, like the object-based landmark task developed here, in order to investigate the temporal and spatial dynamics of the semantic-attention interaction and the process of rebalancing attention after semantic processing with interhemispheric inhibition. For example, the activity of the two FPA networks must be kept in balance in healthy individuals to avoid a unilateral hemispatial neglect syndrome (where one is unable to disengage from one side of visual space (Corbetta & Shulman, 2011). This equilibrium is presumably enabled by the same interhemispheric inhibition that has been widely described (Kinsbourne, 1977; Szczepanski & Kastner, 2013), however, this has yet to be investigated in the context of semantic processing. These investigations should also aim to reveal the commonalities and differences between the processing of overt and covert attention and establish the factors that result in the recruitment of one system over another. Flexible paradigms, using progressively more ecologically relevant stimuli and challenges, will allow for a more detailed assessment of the means (spatial and temporal dynamics) by which other factors that influence the FPA networks and attention allocation exert an effect (see Jewell & McCourt, 2000 for details of other factors).

Our findings represent a vital step forward in our understanding of the mechanisms that underlie the everyday allocation of attention, and the methods used also offer highly sensitive new ways to investigate them. We have recognized and demonstrated that tasks typically used to investigate pseudoneglect in the past have failed to fully encapsulate ecologically relevant variables. To address this, we have utilized paradigms that could accurately reveal the mechanisms supporting the allocation of attention in more complex encounters. For instance, the object-based alternative to the landmark task represents an investigative tool and a template that can be utilized to improve our understanding of the temporal dynamics of attention allocation. This is critical if we are to achieve a complete understanding of how attention is prioritized and guided by external and internal drivers. In turn, this informs the exploration of the neural bases of attention and attention allocation and underpins more sensitive diagnostic techniques for use with patients with brain damage or disease.

## Supplementary Material

10.1037/xhp0000882.supp

## Footnotes

1 Approximation is due to minor variability in each participant’s seating posture.
