# Supplementary material for: A Reassessment of the Pseudoneglect Effect: Attention Allocation Systems Are Selectively Engaged by Semantic and Spatial Processing
Source: J Exp Psychol Hum Percept Perform. 2020 Dec 3;47(2):223–37. doi: 10.1037/xhp0000882 (PMC7818672; doi:10.1037/xhp0000882)
Supplement: Supplementary file 1 [file xhp0000882_supplemental.docx]

**Supplementary Materials 1 – Object-Like Abstract Shape Interpretability**

Methods

*Participants*

Data were collected for 22 (10 males, Mean = 28.41 years old) individuals took part in the online interpretability rating procedure. All participants read the participant information and confirmed their informed consent before at the start of the experimental session. All procedures were approved by the University of Manchester Research Ethics Committee.

*Images*

The online interpretability rating procedure was performed on all object-like abstract shape images (72) used in Experiment 2.

*Procedure*

Participants were presented with each shape and instructed to rate the ease with which they could infer the identity of an object from the image on a 1-5 Likert Scale: 1 – Easy, 2 – Challenging, 3 – Difficult, 4 – Very Difficult, 5 – Impossible. Participants could take as long as they needed to respond and response time was automatically recorded. The order in which shapes were presented was randomised. An example presentation is provided in supplementary figure 1.


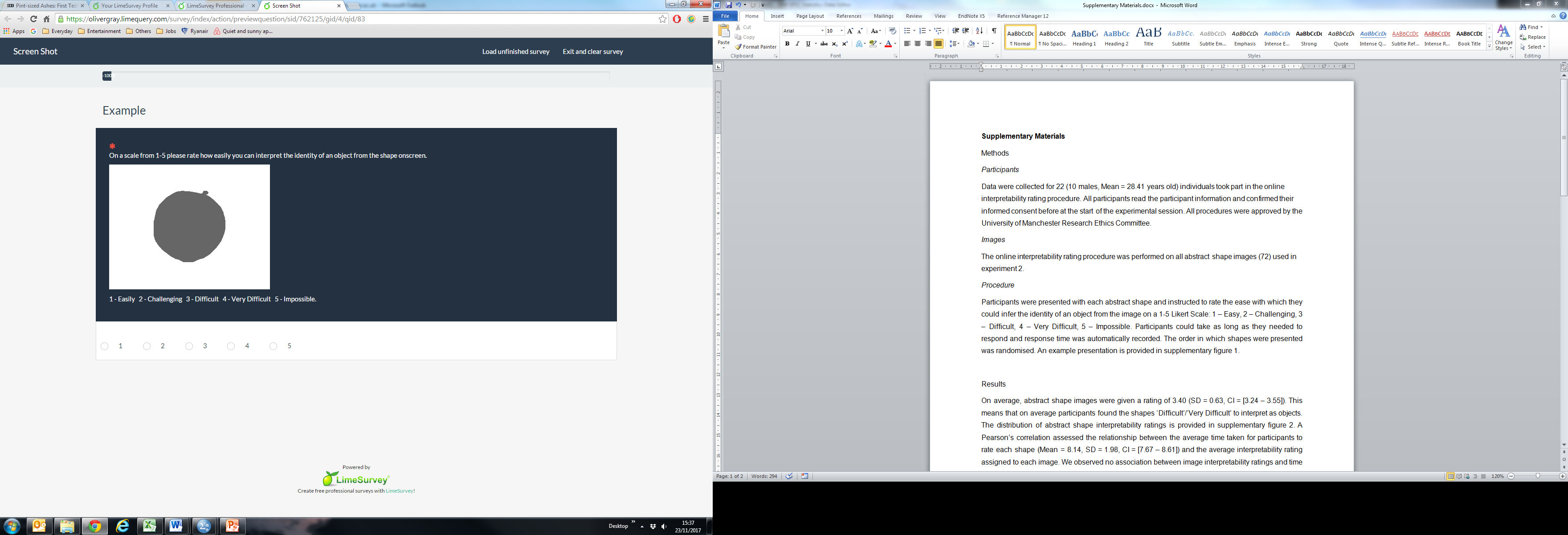


***Supplementary Figure 1:*** *A representative example of the format of the online abstract shape image interpretability rating procedure.*

Results

On average, object-like abstract shape images were given a rating of 3.40 (SD = 0.63, CI = [3.24 – 3.55]). This means that on average participants found the shapes ‘Difficult’/‘Very Difficult’ to interpret as objects. The distribution of abstract shape interpretability ratings is provided in supplementary figure 2. A Pearson’s correlation assessed the relationship between the average time taken for participants to rate each shape (Mean = 8.14, SD = 1.98, CI = [7.67 – 8.61]) and the average interpretability rating assigned to each image. We observed no association between image interpretability ratings and time taken for participants to provide a rating (r = 0.181, p = 0.134).


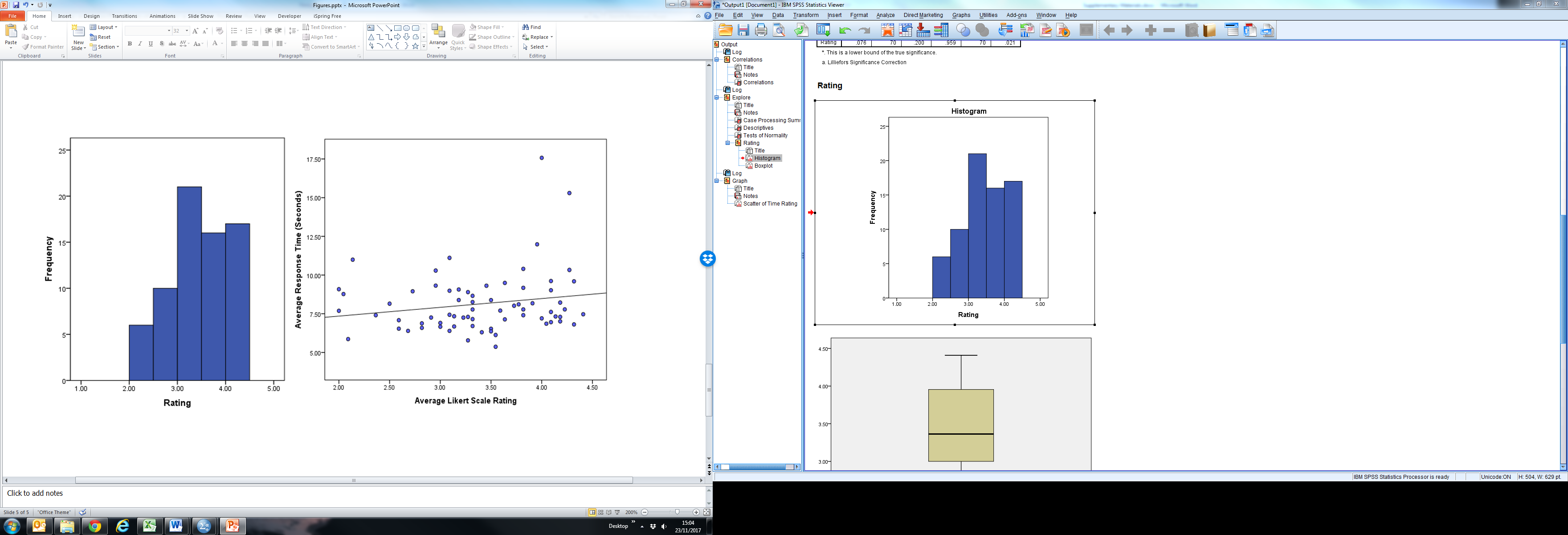

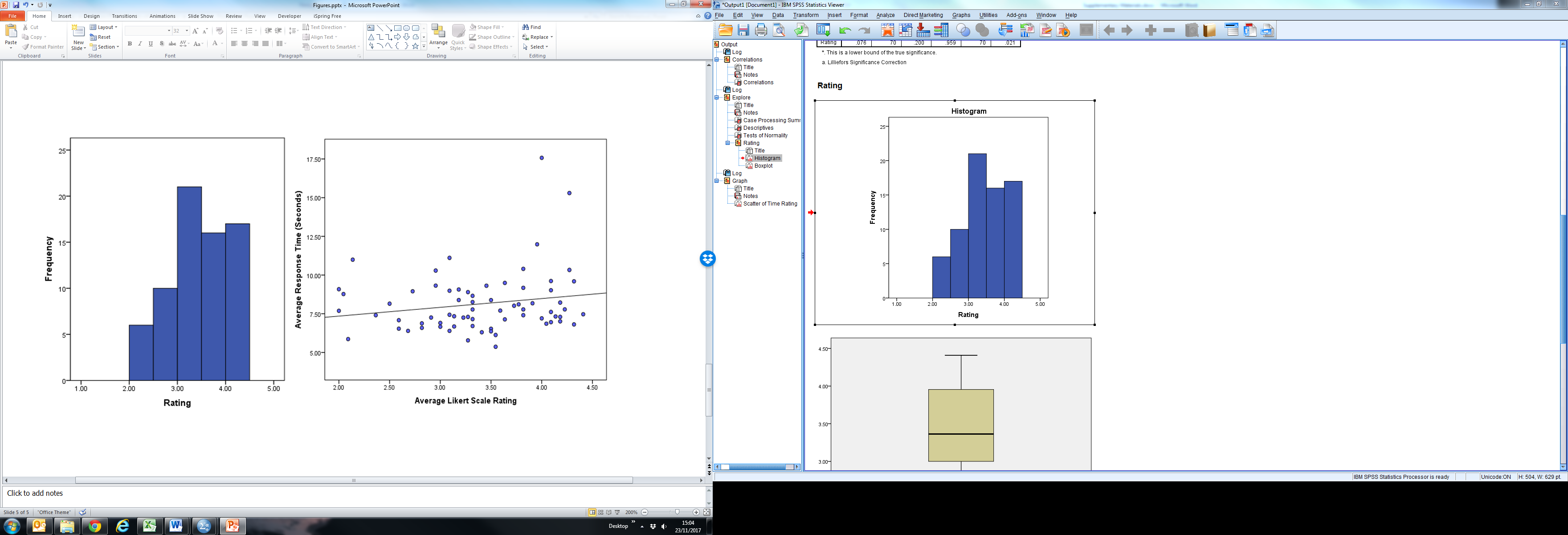


***Supplementary Figure 2: A)*** *A histogram depicting the distribution of the average interpretability rating for each object-like abstract shape image.* ***B)*** *We observed no significant association between abstract shape image interpretability ratings and average response times (r = 0.181, p = 0.134).*
